# Draft Genome Sequence of *Clostridium* sp. Strain FP1, with Similarity to *Clostridium tagluense*, Isolated from Spoiled Lamb

**DOI:** 10.1128/MRA.00321-20

**Published:** 2020-04-30

**Authors:** Nikola Palevich, Faith P. Palevich, Paul H. Maclean, Ruy Jauregui, Eric Altermann, John Mills, Gale Brightwell

**Affiliations:** a AgResearch Ltd., Grasslands Research Centre, Palmerston North, New Zealand; b Riddet Institute, Massey University, Palmerston North, New Zealand; University of Maryland School of Medicine

## Abstract

*Clostridium* sp. strain FP1 was isolated from vacuum-packaged refrigerated spoiled lamb, and this article describes its 5.4-Mb draft genome sequence. The FP1 genome was sequenced to facilitate source tracking and attribution studies, adding to our understanding of the role of *Clostridium* species in premature spoilage of red meats.

## ANNOUNCEMENT

*Clostridium* sp. strain FP1 is a Gram-positive, spore-forming, and slow-growing psychrotrophic anaerobe that was originally isolated from vacuum-packaged refrigerated spoiled lamb at AgResearch Ltd. (Palmerston North, New Zealand). Blown pack spoilage (BPS) is a major issue for the meat industry, and the etiological agents of BPS are numerous members of the *Clostridium* species, including Clostridium estertheticum. FP1 was positive by the C. estertheticum-like real-time PCR used by industry (1) and was selected for genome sequencing to examine its role in BPS (2). Recent amplified ribosomal DNA restriction analysis (ARDRA) carried out on 90 New Zealand psychrotolerant *Clostridium* isolates derived from three meat production animal types and their environments (3) placed strain FP1 into the Clostridium tagluense-like cluster, with >95% similarity to the C. tagluense type strain A121 (4). Here, we present the draft annotated genome sequence of *Clostridium* sp. strain FP1, with similarity to C. tagluense, isolated from spoiled lamb.

Strain FP1 was isolated from the meat drip of vacuum-packaged lamb in 2017 that had no pack distension, with meat discoloration (some green spots) and sweaty feet odor. Meat drip was cultured anaerobically at 10°C in 10-fold suspensions in prereduced peptone-yeast extract-glucose-starch broth (1). Genomic DNA was extracted using a modified phenol-chloroform procedure, as described previously (5). Whole-genome sequencing of FP1 was performed with an Illumina TruSeq Nano library on the Illumina MiSeq platform (2 × 250-bp paired-end reagent kit v2) at the Massey Genome Service (Massey University, Palmerston North, New Zealand). In total, 3,511,896 paired-end raw reads were generated, corrected, trimmed, and *de novo* assembled using the A5-miseq pipeline v20169825 with standard parameters (6). The result of the FP1 assembly was 210 scaffolds with an *N*_50_ value of 126,678 bp, with the largest scaffold being 470,803 bp long. Based on the assembly information, the FP1 draft genome consists of 5,384,978 bp, with a G+C content of 31.0% and 145× coverage. The genomes were annotated with GAMOLA2 (7), DIAMOND v0.9.21.122 (8), InterProScan v5.36-75.0 (9), and OmicsBox v1.1.164 (10) software packages, using default parameters. A total of 5,116 putative protein-coding genes were predicted, along with 97 tRNAs, 45 rRNAs, and 231 noncoding RNA elements.

The dbCAN2 (11) software was used to determine the carbohydrate-active enzyme (CAZy) (12) profile of FP1 using default settings. The FP1 genome encodes a total of 17 glycoside hydrolases, 36 glycosyl transferases, 7 carbohydrate esterases, and 9 carbohydrate-binding protein module families. When compared to the recently sequenced C. estertheticum subsp. *laramiense* strain DSM 14864^T^ (ATCC 51254^T^) (13) and C. estertheticum strain DSM 8809^T^ (ATCC 51377^T^) (14), FP1 encodes one-half as many carbohydrate-active enzymes. We propose that related *Clostridium* species may differ in their abilities to metabolize more complex insoluble polysaccharides, as both FP1 and C. tagluense A121^T^ lack genes encoding polysaccharide lyases and the genes required for uronic acid metabolism.

### Data availability.

The genome sequence data for *Clostridium* sp. strain FP1 were deposited under GenBank accession number JAAMNJ000000000, BioProject accession number PRJNA574489, and Sequence Read Archive (SRA) accession number SRR11113223.
